# Revisiting Nonresidential Environmental Exposures and Childhood Lead Poisoning in the US: Findings from Kansas, 2000–2005

**DOI:** 10.1155/2016/8791686

**Published:** 2016-03-02

**Authors:** Lu Ann Brink, Evelyn O. Talbott, Gary M. Marsh, Ravi Sharma, Stacey Benson, Wen Chi Wu, Chunzhe Duan

**Affiliations:** ^1^University of Pittsburgh, Graduate School of Public Health, Department of Epidemiology, Pittsburgh, PA, USA; ^2^University of Pittsburgh, Graduate School of Public Health, Department of Biostatistics, Pittsburgh, PA, USA; ^3^University of Pittsburgh, Graduate School of Public Health, Department of Behavioral and Community Health Sciences, Pittsburgh, PA, USA

## Abstract

Although blood lead levels (BLLs) in US children have dramatically declined over the past 40 years, there remain pockets of children living in areas with elevated BLLs. While some increases (≥10 *μ*g/dL) may be associated with legacy lead paint, ambient air lead may be contributing to the problem. A deidentified dataset of information on over 60,000 Kansas children under 3 years of age who were tested for BLL was provided through the Kansas Environmental Public Health Tracking Network for the period 2000–2005. Using ArcGIS, we calculated distance (in miles) from a lead-emitting industry referred to as a toxic release inventory (TRI) site. The USEPA TRI database tracks the management of certain toxic chemicals that may pose a threat to human health. US facilities in different industry sectors must report annually amount of substances like lead into the environment including their exact location. Distance from a TRI site was inversely related to BLL after controlling for area-level poverty and pre-1950 housing. The results of our evaluation indicate there is a significant relationship between proximity to lead industry and childhood BLLs. Proximity to sources of lead emissions should be evaluated as a possible factor when identifying children for targeted BLL testing.

## 1. Introduction and Background

Although blood lead levels in US children have dramatically declined over the past 40 years [1], lead poisoning continues to occur. The Lead-Based Paint Poisoning Prevention Act legislation was passed in 1971 and by 1978 the use of lead-based paint in residential housing was banned (http://www.fda.gov/). Regulations phasing out lead in gasoline were implemented in 1973. Although much of the legacy lead paint has been removed from homes, some remains in older homes. The reduction of lead from gasoline and paint has resulted in a decline in US blood lead levels. However, in 2012, there remain significant subgroups of children in both urban and rural areas with elevated blood lead levels [2]. Data evaluated from 26 states that are part of the Centers for Disease Control (CDC) Childhood Lead Poisoning Prevention Program (CLPPP) and made available on the CDC Environmental Public Health Tracking Network (Tracking Network) revealed that almost 95,000 children between 0 and 3 years of age had blood lead levels greater than 10 micrograms per deciliter (*μ*g/dL) from 2000 to 2007 and an estimated 7,000 had elevated blood lead in 2006 alone. In May of 2012, the CDC childhood blood lead action level has been reduced to the top 5% of tests, about equal to 5 *μ*g/dL.

While some of these EBLs may be associated with legacy lead paint or inadequately controlled consumer goods, including consumer products and jewelry, lead in the outdoor environment may also be contributing to the problem [3]. The outdoor environment may be contaminated from residue of leaded gasoline from past decades or from current industrial processes. In 2002, over 9,000 industrial sites in the US reported lead releases to the Environmental Protection Agency (EPA) toxic release inventory (TRI) [4] There are several well-known investigations involving communities near lead smelters and childhood blood lead levels including Dallas, Texas, and Silver Valley, Idaho [5, 6].

Several analyses have shown that air and soil lead levels are higher near industries using lead [7–16]. The US EPA [9] determined that the lead in air surrounding airports can be inhaled directly or may be ingested by children after it settles into the soil; this exposure may influence childhood blood lead levels within 1 km of a source [17]. Two previous studies have shown that a point source of lead is related to elevated childhood blood lead levels for those in closer proximity to the source [18, 19]. Miranda analyzed the blood lead surveillance data from 1995 to 2003 of selected children residing in areas surrounding airports using avgas (a lead-containing aviation fuel), using six counties with and without airports where many children had been screened for lead exposure [18]. The distance of each tested child to an airport was measured using ArcGIS, stratifying to 500, 1000, 1500, and 2000 m. A distance of greater than 2000 m to an airport was considered the reference group. Analyses were controlled for percent older housing, mean income, percent receiving public assistance, and percent minority at the census block level, and season screened. Of the 125,197 children screened in the nine study years, 13,478 were living within 2000 m of an airport. Those living within 500 m had a 4.4% higher mean blood lead, those within 1000 m had a 3.8% higher mean blood lead, and those residing 1500 m had a mean blood lead level that was 2.1% higher than those residing over 2000 m from the airport (*p* < .05).

In 1999, median blood lead levels of children attending the school nearest the Met-Mex Penoles smelter were 27.6 *μ*g/dL [20]. As a result of the elevated blood lead levels, the company was ordered to reduce their emissions and to remediate the environment within 2 km of the plant. Soto-Jiménez and Flegal [19] evaluated environmental samples (including aerosols, soils, and outdoor and indoor dust) and blood samples of 34 children aged 2–17 living within 2 km of a smelter in Torreon, Mexico. After controlling for household exposures, including cleanliness and recent renovations to the home, as well as previous blood lead levels, they found no relationship between proximity to the smelter and blood lead level. However, they did not sample residents more than 2 km from the site as a control population.

Childhood lead data available on the CDC Environmental Public Health Tracking Network enabled us to conduct a linkage study of the relationship between airborne lead and the prevalence of elevated childhood blood lead levels at the county level in the US, controlling for the proportion of older (pre-1950) housing, potentially containing leaded paint [20]. A total of 1508 counties were included in the analysis which spanned 2000–2007 representing 5,047,619 children. In this ecologic analysis, modeled ambient air lead levels from the EPA National-Scale Air Toxics Assessment (NATA) were used to estimate residential exposure to lead. The National Air Toxics Assessment was conducted by EPA in 2002 to assess air toxics emissions in order to identify and prioritize air toxics, emission source types, and locations which are of greatest potential concern in terms of contributing to population risk [21]. This data source provides downloadable information on emissions at the state, county, and census tract level. A geospatial regression (*R* ^2^ = .342, *p* < .001) revealed that air lead, percent of older housing, and poverty were all significant predictors of percent elevated. In multivariate negative binomial regression, NATA modeled air lead was a significant predictor of childhood blood lead (% ≥10 *μ*g/dL) after controlling for percent of pre-l950 housing, rural classification, and percent of African Americans by county.

Our evaluation of the relationship of airborne lead and elevated blood lead at the county level for the US indicates that there may be a significant relationship between ambient air lead and childhood blood lead levels, controlling for older housing. The results of our county-level study highlight the need to be aware of not only lead paint in the home, but also nearby industrial lead emissions when evaluating the risk for elevated blood lead.

Although our ecological analysis has shown a relationship between county-level airborne lead levels and proportion of children with elevated BLLs in the nation, this relationship was not clear in Kansas. However, those counties with any child with a BLL over 10 *μ*g/dL had significantly higher air lead estimates than those without an elevated BLL child.

The more important measure in a rural area may be distance from an industrial process. Proximity to industry may indicate legacy and/or fugitive lead emissions from the industry, which may deposit on or near the property. Contaminated soil near this industry may serve as a source of lead for young children living nearby. Although pre-1950 housing potentially containing lead paint is arguably the most important source of lead exposure to a young child, close proximity to a lead industry is also a significant predictor of individual BLL.

The current effort furthers this at a more refined geographic level. To more fully understand the potential contribution of nonresidential environmental lead and its concomitant risk from lead ingestion or inhalation in small children, we have continued this analysis with our partner Tracking States with individual childhood blood lead levels.

## 2. Methods

### 2.1. Population Background

Kansas is a Midwestern state in the middle of the US with a population as of the 2010 census of 2,904,021. The racial makeup of the population was 83.8% white American and 5.9% African American. Ethnically 10.5% of the total population was of Hispanic or Latino origin. A total of 9.5% of its inhabitants live below the poverty level. It ranks 40th in population density with 13 counties in more urban areas making up a population of 1,593,706 and the remaining 92 counties comprising 941,341 in population.

Although it is well known for its agricultural outputs including transportation equipment, commercial and private aircraft, lead battery plants, food processing, cattle, sheep, wheat, soybeans, hogs, corn, and salt, there is an industrial presence that includes publishing, chemical products, machinery, apparel, petroleum, and mining. It has an overall proportion of pre-1950 housing of 23.4% and has 27 toxic release inventory sites that emit more than 500 pounds of lead. These are also primarily near the more urban counties where industry is also centered. Saline County, Kansas, has been the subject of a specific study for air lead and blood lead as an Exide Technologies lead battery plant, one of the world's largest, had been designated a nonattainment area for the National Ambient Air Quality Standard for lead in 2010-2011 [22].

### 2.2. Individual Blood Lead Data

A deidentified dataset of over 60,000 Kansas children under 36 months who were tested for blood lead was provided through the Kansas Environmental Public Health Tracking Network for 2000–2005. Geocoordinates were available on more than 40,000 children. Individual level data for the child included blood lead level, age in months, and race. Using ArcGIS, we calculated distance (in miles) from a lead-emitting TRI site and distance (in miles) from a nonlead TRI site (in miles). Census tract level data included percent pre-50 housing, poverty rates, and rural percentage within each county. Univariate analysis of blood levels by distance from a TRI facility was conducted using SPSS 20.0. Both individual level and tract level data were entered into a multilevel random intercept model and analyzed in *R* [23]. A multilevel analysis includes variability at each level (individual level and census tract level) of nesting that is usually not accounted for in a multiple regression analysis.

Every three years since 1996, EPA has compiled a national-scale air toxics assessment (NATA). The assessment is a state-of-the-science tool that provides estimates of the concentrations, exposures, and broad estimates of the risk from breathing air toxics. In 2002, NATA evaluated 180 of the 187 air toxics, including lead and lead compounds. The 2005 NATA toxics used an emissions inventory of outdoor stationary and mobile sources (National Emissions Inventory, NEI). This includes major stationary sources, for example, large waste incinerators to provide estimates of 177 of the 187 air toxics sources. The NATA assessment involves compiling lead levels from airport point sources as well as emissions from smaller establishment, for example, dry cleaners, small manufacturers, wildfires and both on-road and nonroad mobile sources, for example, cars, trucks, planes, and boats.

We used modeled ambient concentrations of lead and lead compounds at the census tract level for the 2005 National-Scale Air Toxic Assessment (NATA) downloaded from the EPA website [24]. There were notable improvements from the 2002 to 2005 NATA estimates, which included 19,000 airports as point sources, on-road and nonroad inventories were updated, and the state-of-the-art Community MultiScale Air Modeling (CMAQ) model was used. Because of these improvements, the 2005 model was used. These air lead levels were merged by FIPS code to the county of interest and its corresponding blood lead testing data. The FIPS code is an abbreviation for Federal Information Processing Standards. These are standard codes for a county or census tract to identify a specific geographic area within the US [25]. They were developed for use in computer systems by nonmilitary government agencies and contractors and are used extensively to link geographic and demographic information in Geographical Information Systems.

### 2.3. 2000 Census Data

Additional census tract variables that were downloaded to explore their predictive value for childhood blood lead levels include percentage of older (pre-1950) housing, percent living in poverty, and racial makeup of the county. The three types of data (health, EPA NATA, and census) were merged by FIPS code using SPSS. Poverty was measured using the census definition which is a set of money income thresholds that vary by family size and composition to determine who is in poverty. If a family's total income is less than the family's threshold, then that family and every individual in it are considered in poverty [26].

## 3. Results

The data included all blood lead measurements for 2000–2005 within the 105 contiguous counties within Kansas for children 0–36 months. There were an average of 120,000 children under the age of three years residing in Kansas during 2000–2005 and as shown in Table 1, of those tested, 11.6% had BLL over 5 *μ*g/dL and 3.3% were ≥10 *μ*g/dL, until recently considering the action level. Testing for blood lead increased yearly, from 1,878 tested in the year 2000 to 11,206 tested in 2005. The gender distribution of tested children was 20,653 (50.1%) males, 19,810 (48.0) females, and 765 (1.9%) unknown. The average BLL decreased annually, from 4.6 in 2000 to 3.6 in 2005. The proportion with more than 10 *μ*g/dL decreased from 9.6% in 2000 to 2.3% in 2005. Similarly, those with BLL within 5–9 *μ*g/dL decreased from 14% to 10.2% in the same time period.

**Figure 1:**
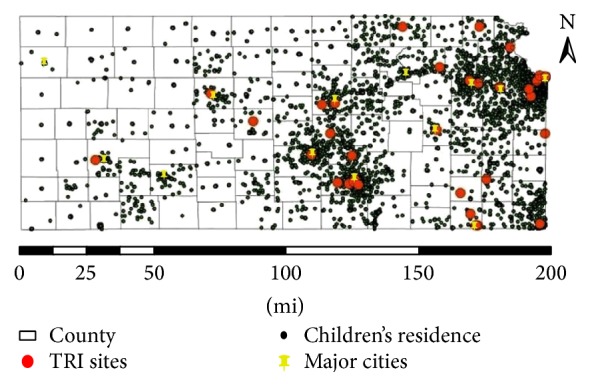
Proximity of TRI sites to major cities and children's residence in Childhood Lead Screening Study in Kansas, 2005–2010.

Average blood leads went down from 2000–2005 as the number of children tested increased. In 2000, 1,878 children were tested and in 2005 11,206 were tested. This most likely represents an increase in efforts of the screening program. The overall average BLL for the 41,228 children aged 0–36 months with a geocode was 3.81 *μ*g/dL and for those without a geocode was 3.32 *μ*g/dL. Over 41% of the geocodes were missing although this was reduced over time. It is not possible to comment on the reasons for the lower mean average because for over half of the nongeocoded participants, there was no county, zip code, census tract, or any indication of a rural route delivery. This makes it just as likely that the children were in inner city as rural. See Figure 1 (proximity of geocoded participants in the childhood blood screening program and proximity to Lead TRI sites and major cities in Kansas 2000–2005).

Among the thirteen counties with a resident child with a blood lead over 10 *μ*g/dL, the mean NATA lead concentration was 0.00177 and among those counties with no children with blood lead over 10 *μ*g/dL, the mean NATA lead concentration was 0.00064, a 2.77-fold greater lead concentration in counties with an elevated blood lead child. The *t*-test for this difference in airborne lead concentration was significant (*p* < .0001). However, no relationship between NATA air lead estimates and mean blood lead by census tract or county was found.

We compared blood lead levels with proximity (miles) to a lead TRI site. While both mean BLL and proportion elevated decreased with increasing distance from a TRI site, so did pre-1950 housing and poverty, potentially explaining the decrease in BLL (see Table 2). Therefore, we conducted an identical analysis of distance from a nonlead TRI site (toluene). Although poverty and pre-1950 housing decreased again, blood lead levels had no discernible pattern. The slope of the blood lead by decreasing distance to a Lead TRI site was significant for both mean blood lead and percent with elevations in ≥10 *μ*g/dL Pb lead levels. This was not evident in Table 3 for proximity to a toluene site.

Although other risk factors for elevated blood lead, including both poverty and pre-50 housing, are highest near both lead and toluene TRI sites, BLLs are highest closest to the lead TRI sites. The test for a significant slope (linear) was not significant for proximity to a toluene TRI site.

To control for older housing and poverty, we created a multilevel model to predict BLL using distance from a lead TRI site, child's age in months, and poverty and pre-1950 housing at the census tract level. For the model with a lead emitting TRI site within 10 miles, the coefficient of distance becomes negative (higher BLL with decreasing distance) and all covariates are significant. The smaller the distance from the TRI site, the higher the child's blood lead level. Given the parameterization of the model, the coefficient for* dist_mile*, ${\hat{\beta }}_{1}=-.010$, represents the relationship of average level of log blood lead to TRI distance, after controlling for pre-1950 housing and poverty (see Table 4). The coefficients reflect one-month change for age, a percentage point change for percent poverty, and pre-fifty housing and a one microgram/deciliter change in blood lead level for each mile distance from a TRI site; for example, the greater the distance from lead TRI site, the greater the reduction in blood lead level.

## 4. Discussion

This was a semiecological study in that we had exact location of the home, the age of the child in months, and the child's blood lead level; however percent pre-1950 housing and percent poverty were US census estimates measured at the census tract level. The results of our evaluation of the relationship of proximity to lead industry and blood lead levels in Kansas indicate that after controlling for percent pre-1950 housing, age of child, and percent poverty there was a significant relationship between proximity to lead industry and childhood blood lead levels. This is especially important as many of the typical risk factors for childhood blood lead have been removed (including leaded gasoline and paint). The results of this study show an association between proximity to industry and not only absolute lead level but also proportion of children elevated among those screened in Kansas within 2000–2005. Although we still lack individual level information on age of the child's home, we were able to control for proportion of older homes within the child's census tract of residence. Further analysis of proximity to a nonlead industry helped to reinforce our findings, as this appears to be more than an environmental justice issue of poverty and older housing near industrial sites.

Potential biases were the inability to geocode all of the residential information on the children screened with a match rate of 60 percent. Vine et al. [27] found that match rates varied from 20% in rural counties to 98% in urban counties. Because county was not available if geocode was not available, we were not able to assess whether there were urban and rural differences in our match rates. If the match rates were similar to those of Vine, our results may be more generalizable to urban children. A scatterplot of the children who came in for screening however clearly shows a preponderance of children clustered closer to the major cities of Spokane, Topeka, Kansas city, and Wichita as well as to the majority of the TRI lead sites within the state (see Figure 1). There is also the potential for response bias by year as the early years of this program (2000–2002) had far few children screened for blood lead levels.

Although we also considered air lead levels modeled as part of a national air toxics assessment program by the USEPA (NATA) in 2005, there appeared to be no consistent relationship with TRI distances. This was census tract of residence modeled data which was used and may not have been sensitive or specific to year of testing to capture specific residential ambient air exposures. NATA is modeled only every three years at the census tract level.

Proximity to sources of lead emissions should be evaluated as a possible factor to include when identifying children for targeted testing and when evaluating the home environment of a child with elevated blood lead levels. Future research could verify the significance of this study by connecting the source to the blood lead levels. Rosman has suggested choosing a site with a recent release of lead that has a distinct lead isotope and examining the isotope ratios in the blood as one moves away from the source. This would separate legacy lead from recent emissions [28].

## Figures and Tables

**Table 1:** Annual number of children aged 0–36 months tested for blood lead levels in Kansas within 2000–2005 (geocoded only 41,228).

	2000	2001	2002	2003	2004	2005	Total
Geometric mean (CI)	3.40 (3.28, 3.52)	3.09 (3.02, 3.17)	3.25 (3.19, 3.31)	3.08 (3.04, 3.12)	3.16 (3.12, 3.19)	3.07 (3.04, 3.10)	3.13 (3.12, 3.15)
Arithmetic mean (CI)	4.60 (4.40–4.80)	4.05 (3.93, 4.17)	4.20 (4.10, 4.29)	3.69 (3.64, 3.74)	3.69 (3.65, 3.74)	3.65 (3.60, 3.69)	3.81 (3.79, 3.84)
<5 *μ*g/dL	1436 (76.5%)	2810 (81.5%)	3943 (78.1)	7606 (86.6%)	9486 (87.3%)	9804 (87.5%)	35085
5 to 9 *μ*g/dL	262 (14.0%)	410 (11.9%)	824 (16.3%)	961 (10.9%)	1165 (10.7%)	1143 (10.2%)	4765 (11.6%)
10+ *μ*g/dL	180 (9.6%)	228 (6.6%)	284 (5.6%)	215 (2.4%)	212 (2.0%)	259 (2.3%)	1378 (3.3%)

Total tested	1878	3448	5051	8782	10863	11206	41228

**Table 2:** Descriptive characteristics of population by distance from lead TRI site of reported annual emissions in excess of 500 pounds.

Distance from TRI site (miles)	Number of children	Mean BLL^*∗*^	95% confidence interval for mean		Mean% pre-50 housing	NATA 2005 air lead estimates (*∗*10^−3^) *µ*g/m^3^	Mean% poverty	Percent with blood lead ≥ 10 *μ*g/dL^*∗∗*^
			Lower bound	Upper bound				
0 to 0.25	99	5.01	3.87	4.40	65.00	1.28	18.34	11.11
0.26 to 0.5	355	4.56	4.08	4.41	67.80	1.50	18.58	7.89
0.51 to 0.75	590	4.50	3.91	4.14	70.05	2.00	19.51	5.93
0.76 to 1	1028	4.47	3.79	4.06	70.86	2.52	21.86	6.32
1 to 1.99	4398	4.14	3.85	3.97	63.52	3.64	16.77	4.18
2 to 2.99	4649	3.87	3.68	3.81	56.53	2.59	15.33	3.48
3 to 3.99	4598	3.85	3.26	3.42	53.45	1.93	16.15	3.45
4 to 4.99	2762	3.72	3.46	3.64	44.93	1.61	14.11	2.82
5 or more	22749	3.68	3.80	3.90	40.26	0.91	9.84	2.88

^*∗*^Slope significant (*p* < .001).

^*∗∗*^Slope significant (*p* < .005).

**Table 3:** Descriptive characteristics of population by distance from toluene emitting TRI site.

Distance from TRI site (miles)	Number of children	Mean BLL	95% confidence interval for mean		Mean% pre-50 housing	NATA 2005 air toluene estimates (*∗*10^3^) *µ*g/m^3^	Mean% poverty	Percent with blood lead ≥ 10 *μ*g/dL
			Lower bound	Upper bound				
0 to 0.25	88	3.72	3.05	4.40	52.82	.340	33.52	3.41
0.26 to 0.5	331	4.21	3.85	4.58	54.88	.146	28.71	5.44
0.51 to 0.75	468	3.88	3.64	4.11	53.86	.086	23.36	3.42
0.76 to 1	1005	3.95	3.77	4.13	55.80	.075	24.72	3.68
1 to 1.99	6587	4.06	3.98	4.13	59.13	.030	25.18	4.16
2 to 2.99	4926	3.58	3.51	3.66	49.38	.026	19.44	2.56
3 to 3.99	2631	3.36	3.57	3.45	41.44	.032	17.65	1.90
4 to 4.99	2035	3.34	324	3.44	30.59	.015	12.65	1.97
5 or more	23157	3.88	3.84	3.91	46.03	.012	14.65	3.52

**Table 4:** Results of multilevel model predicting childhood blood lead level in KS.

Variable	Coefficient (*β*)	Standard error	*Z*	*p* value	95% CI
Miles from TRI site	−0.010	0.004	−2.69	.007	−0.017 to −0.003
Age	0.010	0.000	22.73	<.001	0.009 to 0.011
% poverty	0.006	0.001	5.15	<.001	0.004 to 0.008
% pre-50	0.003	0.000	7.71	<.001	0.002 to 0.003
Constant	0.768	0.029	26.39	<.001	0.711 to 0.825
